# The Possible Mechanisms Involved in Degradation of Patulin by *Pichia caribbica*

**DOI:** 10.3390/toxins8100289

**Published:** 2016-10-09

**Authors:** Xiangfeng Zheng, Qiya Yang, Hongyin Zhang, Jing Cao, Xiaoyun Zhang, Maurice Tibiru Apaliya

**Affiliations:** School of Food and Biological Engineering, Jiangsu University, 301 Xuefu Road, Zhenjiang 212013, Jiangsu, China; 1879600292l@163.com (X.Z.); yangqiya1118@163.com (Q.Y.); caojing819@126.com (J.C.); zhangxiaoyungu@126.com (X.Z.); mtapaliya@yahoo.com (M.T.A.)

**Keywords:** mycotoxin, patulin, biodegradation, *Pichia caribbica*, proteomics, intracellular and extracellular enzymes

## Abstract

In this work, we examined the mechanisms involved in the degradation of patulin by *Pichia caribbica*. Our results indicate that cell-free filtrate of *P. caribbica* reduced patutlin content. The heat-killed cells could not degrade patulin. However, the live cells significantly reduced the concentration of the patulin. In furtherance to this, it was observed that patulin was not detected in the broken yeast cells and cell wall. The addition of cycloheximide to the *P. caribbica* cells decreased the capacity of degradation of patulin. Proteomics analyses revealed that patulin treatment resulted in an upregulated protein which was involved in metabolism and stress response processes. Our results suggested that the mechanism of degradation of patulin by *P. caribbica* was not absorption; the presence of patulin can induce *P. caribbica* to produce associated intracellular and extracellular enzymes, both of which have the ability to degrade patulin. The result provides a new possible method that used the enzymes produced by yeast to detoxify patulin in food and feed.

## 1. Introduction

Phytosanitation is critical in food safety in the globalized agribusiness, where fresh fruits have been considered as promising natural food. The Food and Agricultural Organization (FAO) estimated that 25% of the world’s crops are contaminated with mycotoxins, and *Aspergillus*, *Penicillium*, and *Fusarium* genera were incriminated [1,2,3]. Patulin (4-hydroxy-4H-furo [3,2c] pyran, 2[6H]-one) one of these mycotoxins is an unsaturated heterocyclic lactone produced by certain fungi species (*Penicillium*, *Aspergillus*, and *Byssochlamys*). Patulin is the most common mycotoxin found in apples and its derived products [4]. *Penicillium expansum* is the most common fungus that causes blue mold and patulin contamination in stored apples [5]. Patulin was first isolated from *Aspergillus clavatus* and studied in the early 1940s [6,7]. Patulin contamination is a world-wide problem including, Portugal [8,9], Belgium [10], India [11], Northeast China [12]. Patulin has been demonstrated to induce oxidative stress and causes DNA strands to break in HepG2 cells. Oxidative damage in human cells can lead to mutagenic [13], carcinogenic [14], immunotoxic [15], neurotoxic [16], genotoxic, and teratogenic [17,18] effects.

Traditionally, to control blue mold and patulin contamination in apples, synthetic fungicides are usually relied upon. However, the development of fungicide resistance by pathogens and the public’s concern over the presence of chemical residues in food have prompted an urgent need for alternative control with good efficacy, and little or no toxicity to the non-target organisms [19]. In recent years, biological control using antagonistic yeasts has emerged as a promising method to reduce synthetic fungicides [20]. In view of this, many researches have shown that some antagonist yeasts could directly inhibit the production of patulin. Through almost 30 years of research, dozens of yeasts, including *Pichia caribbica* [21], *Rhodotorula glutinis* [22], *Pichia ohmeri* [23], *Rhodosporidium kratochvilovae* [24], *Gluconobacter oxydans* [25], and several others have been shown to degrade patulin, and inhibited the growth of *P. expansum*. Coelho et al. [26] reported that patulin concentration of 223 µg was decreased over 83% by *P. ohmeri* 158 cells when incubated at 25 °C for two days at a humidity >99. Also, *R. glutinis* and *Cryptococcus laurentii* degraded patulin in vivo and significantly reduced the accumulation of patulin in apples [22]. Zhu et al. [27] reported that the yeast *Rhodosporidium paludigenum* reduced the patulin content in apples.

However, the mechanism(s) of action of yeasts is/are insufficient and remain poorly understood. A study by Coelho, Celli, Ono, Wosiacki, Hoffmann, Pagnocca, and Hirooka [26] showed that antagonistic yeast cells incubated with PAT decreased its contamination through two mechanisms: (1) PAT adsorption at the yeast cell wall, and (2) PAT absorption into yeast cells. Zhu et al. [28] pointed out that, when dead and live cells of *Rhodosporidium paludigenum* were incubated with patulin for three days, 51% of the patulin was absorbed by the dead cells while no patulin was detected in the viable yeast cells.

Genome-wide analysis of the model yeast *Saccharomyces cerevisiae* exposed to patulin indicated that there were upregulated genes which showed proteasome activity, metabolism of sulfur amino acids, and stress responses [29]. Ianiri et al. [30] reported that patulin was degraded by *Sporobolomyces* sp. strain IAM 13481 and two kinds of mesostate were produced. To determine the genes responsible for the degradation, 3000 mutants were instructed by T-DNA insertion, and some were proven to be sensitive to patulin. The genes which include *YCK2*, *PAC2*, *DAL5,* and *VPS8* were annotated to *Saccharomyces cerevisiae*. Ianiri et al. [31] described a transcriptomic approach based on RNAseq to study the changes of gene expression in *Sporobolomyces* sp. exposed to patulin. In their study, the upregulated *Sporobolomyces* genes were those involved in metabolic processes, oxidation-reduction, and transport processes. The patulin decreased the expression of genes involved in the processes of protein synthesis, modification, cell division, and cell cycle. The results provide a comprehensive analysis to identify potential mechanisms and enzymes that are involved in patulin degradation. However, there were no genes and enzymes found to be responsible for the patulin degradation.

In vitro test indicated that *P. caribbica* can degrade patulin directly [21]. However, the mechanism of degradation of patulin by the *P.* *caribbica* is unknown. Therefore, in the present study, we investigated the possible mechanisms involved in degradation of patulin by *Pichia caribbica* and provided a comprehensive analysis of the genes and enzymes that are involved in patulin degradation.

## 2. Results

### 2.1. Effect of Cell-Free Filtrate of P. caribbica on Patulin Degradation

**Figure 1:**
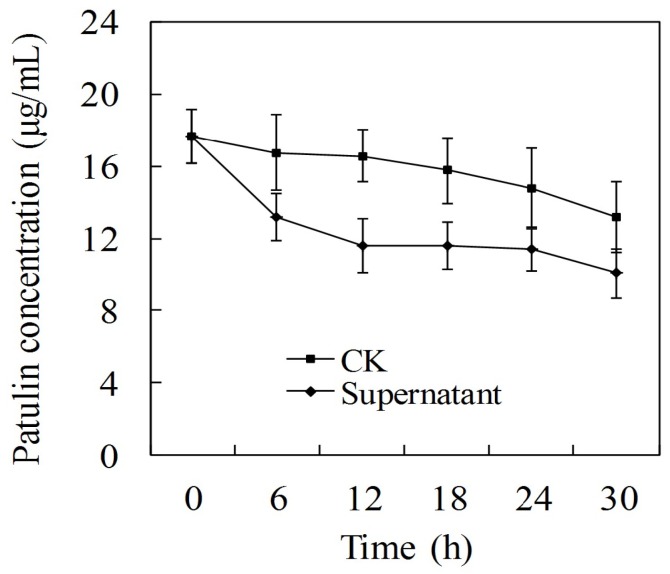
Efficacy of cell-free filtrate of *P. caribbica* on the degradation of patulin. The *x* axis represents the time after the addition of patulin (h: hour), the *y* axis represents the concentration of the patulin in the medium. CK: NYDB + patulin, Supernatant: *P. caribbica* cultuered in NYDB medium + patulin. Results are presented as means ± SD of triplicate experiments. The data at the same time were analyzed by the *t* test. The significant difference was assessed at the level *p* < 0.05.

The findings revealed that the cell-free filtrate of *P. caribbica* reduced the patulin concentration compared to the control (CK) during all the tested time, Figure 1. This demonstrates that that the cell-free filtrate significantly affected the patulin content. The patulin content was reduced from 18.8 μg/mL at 0 h to 11.4 μg/mL at 12 h after incubation, however, after 12 h the degradation declined steadily throughout the entire duration.

### 2.2. Effect of Viable and Heat-Killed P. caribbica Cells on Patulin Degradation

**Figure 2:**
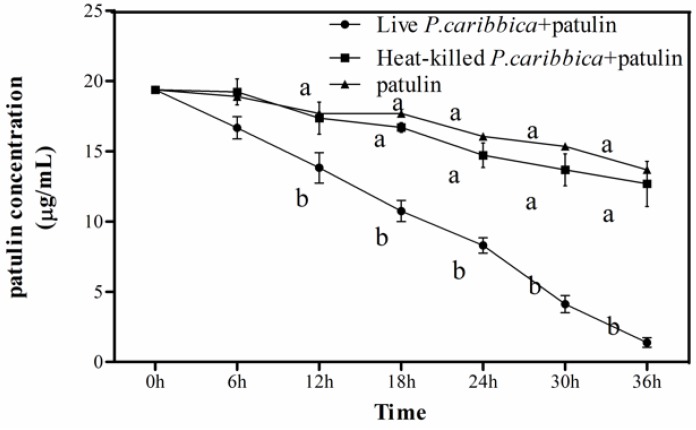
Efficacy of viable and heat-killed *P. caribbica* cells on degradation of patulin. The *x* axis represents the time after the addition of patulin (h: hour), the *y* axis represents the concentration of the patulin in the medium. Results are presented as means ± SD of triplicate experiments. The data at the same time were analyzed by the analysis of variance (ANOVA) in the statistical program SPSS/PC version 17.0. The significant difference was assessed at the level *p* < 0.05.

The assay demonstrated that compared to the control, the dead yeast cells could not degrade the patulin, while the live yeast cells significantly reduced the patulin level throughout the 36 h inoculation period, Figure 2. The live *P. caribbica* cells decreased the patulin concentration to 1.4 μg/mL at 36 h of incubation. However, the patulin concentration of the control and the heat-killed cells were 13.7 μg/mL and 12.7 μg/mL at 36 h after incubation, respectively.

### 2.3. Degradation of Patulin by P. caribbica Was Inhibited by Cycloheximide

**Figure 3:**
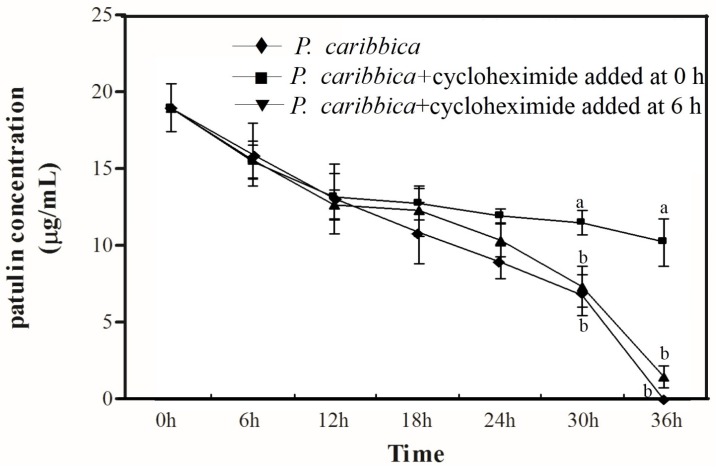
Effects of cycloheximide on degradation of patulin by *P. caribbica*. The *x* axis represents the time after the addition of patulin (h: hour), the *y* axis represents the concentration of the patulin in the medium. Results are presented as means ± SD of triplicate experiments. The data at the same time were analyzed by the analysis of variance (ANOVA) in the statistical program SPSS/PC version 17.0. The significant difference was assessed at the level *p* < 0.05.

The results on the treatment without cycloheximide (*P. caribbica* alone) decreased the patulin level completely after 36 h of incubation, Figure 3. However, the cycloheximide that was added to the patulin at the beginning of the experiment decreased slightly and declined steadily. The patulin content in which the cycloheximide was added at after 6 h of incubation followed a similar trend with that of the control.

### 2.4. Effect of P. caribbica’s Supernatant on Patulin Degradation

**Figure 4:**
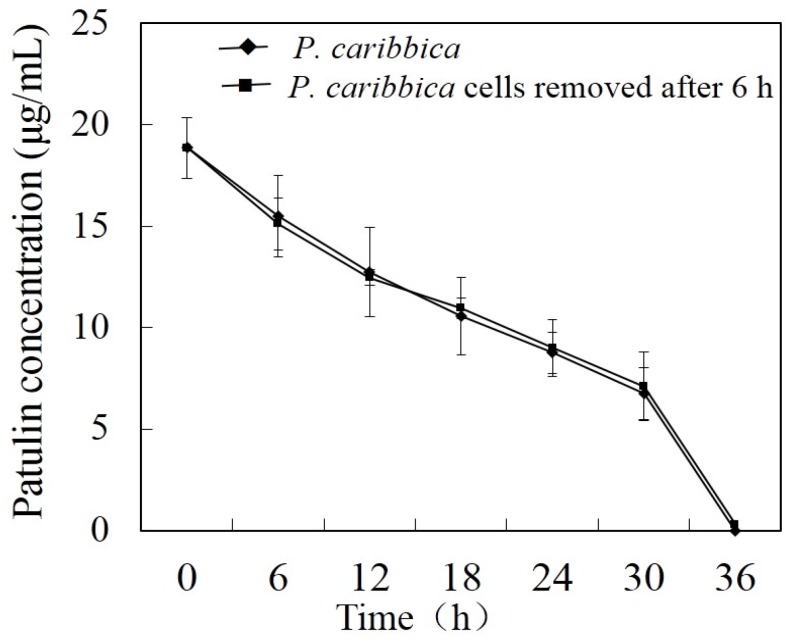
Efficacy of cell-free filtrate of *P. caribbica* which was induced 6 h by patulin on degradation of patulin. The *x* axis represents the time after the addition of patulin (h: hour), the *y* axis represents the concentration of the patulin in the medium. Results are presented as means ± SD of triplicate experiments. The data at the same time were analyzed by the *t* test. The significant difference was assessed at the level *p* < 0.05.

The supernatant of *P. caribbica* significantly reduced the patulin content, regardless of whether the yeast cells were filtered after 6 h or not, Figure 4. The supernatant without yeast cells had no effect on patulin degradation. Henceforth, no significant difference was observed between the two treatments.

### 2.5. Effect of Intracellular Enzymes of P. caribbica on Patulin Degradation

**Figure 5:**
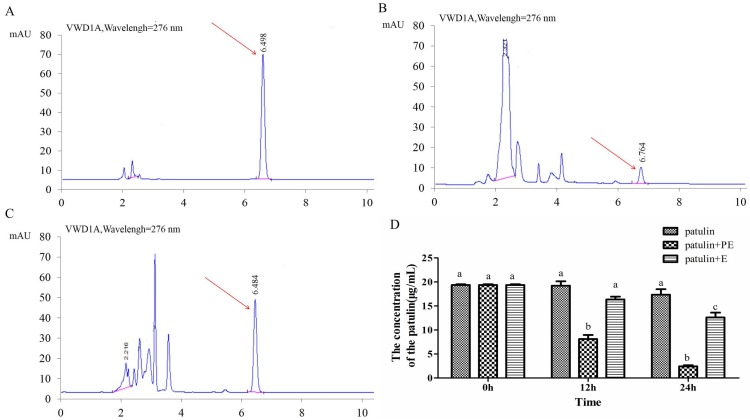
Effects of intracellular enzymes of *P. caribbica* on degradation of patulin. (**A**): The HPLC result of standard patulin samples in phosphate buffer at 24 h after incubation; (**B**): The HPLC result of patulin+P-E (extracted from the *P. caribbica* induced by patulin);(**C**): The HPLC result of patulin+E (extracted from *P. caribbica*); (**D**): The patulin content at 0, 12, and 24 h after treatment. The red arrows in **A**, **B*,*** and **C** represent the peaks of patulin. The data are presented as means ± SD of triplicate experiments. The data at the same time were analyzed by the analysis of variance (ANOVA) in the statistical program SPSS/PC version 17.0. The significant difference was assessed at the level *p* < 0.05.

The results on the intracellular enzymes demonstrates that the intracellular enzymes obtained from the *P. caribbica* that were incubated in the NYDB did not exhibit degradation effect. The patulin content remained at a high level of 8.3 μg/mL at 24 h after incubation, Figure 5C,D. However, the intracellular enzymes from *P. caribbica* incubated in the NYDB and amended with the patulin degraded the patulin to 0.25 μg/mL at 24 h after incubation, Figure 5B,D.

### 2.6. Identification of Differentially Expressed Proteins

**Figure 6:**
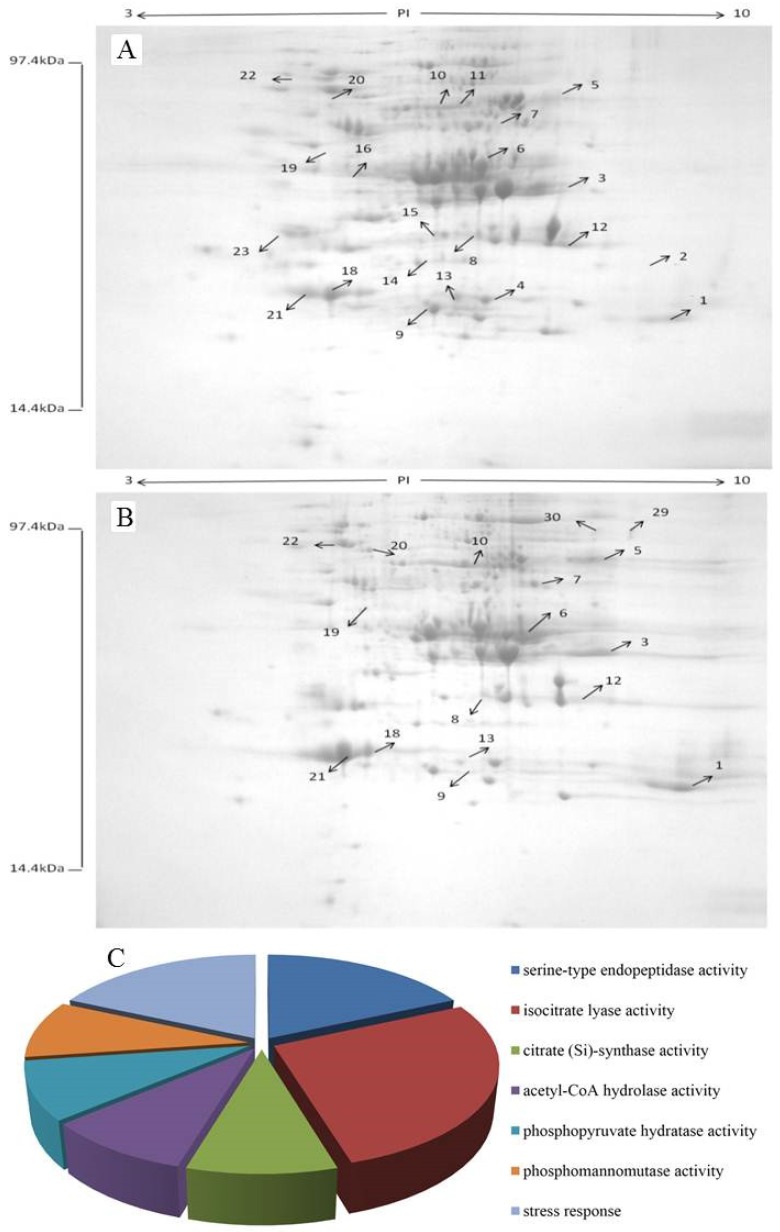
Two-dimensional pattern of intracellular proteins of *P. caribbica* after cultivation for 24 h in NYDB and NYDB amended with patulin. (**A**): Protein extracted from *P. caribbica* which was harvested from NYDB at 24 h after incubation; (**B**): Protein extracted from *P. caribbica* which was harvested from NYDB amended with patulin at 24 h after incubation; (**C**): Gene ontology (GO) analysis of the differentially expressed proteins of *P. caribbica* when treated with patulin.

Proteins were identified by means of peptide mass fingerprints (PMF). MASCOT was used to search protein database of Viridiplantae. More than 150 protein spots were detected in each gel after ignoring very faint spots and spots with undefined shapes and areas using Image Master 2D Elite software, Figure 6A. Protein was extracted from *P. caribbica*, harvested at 24 h after incubation in NYDB, Figure 6A, and NYDB amended with patulin, Figure 6B. A total of 53 differentially expressed proteins were identified from the *P. caribbica* harvested from the NYDB amended with patulin. Out of the 53 proteins, 18 proteins were upregulated and 35 proteins were downregulated. Our test focused on 27 of them, Table 1. The differentially expressed proteins were classified with GO analysis, Figure 6C. Most of the differentially expressed proteins were related to basic metabolism such as isocitrate lyase activity, citrate (Si)-synthase activity, acetyl-CoA hydrolase activity, phosphomannomutase activity, and phosphopyruvate hydratase activity, Figure 6C. This indicated that the basic metabolism of *P. caribbica* was activated by patulin. The responses of *P. caribbica* to patulin are complex, as the differentially expressed proteins were involved in multiple metabolic pathways. Heat-shock protein 70 and Heat-shock protein SSB1 under the category of stress response may be related to the yeast cell’s stress response to the patulin, Figure 6C.

## 3. Discussion

Patulin is a worldwide food mycotoxin which is present in a variety of fruit products. In recent years, attempts have been made to reduce the mycotoxin content in fruits and their derived products [32]. In view of this, biodegradation of mycotoxins has taken center stage for many researchers. Some antagonistic yeast strains used in biological control of fruits do not only control pathogens in the storage period, but also reduce patulin and other mycotoxins directly. Our research team found that the degradation of patulin treated with *P. caribbica* was higher than that of the control at all the tested time, which showed that *P. caribbica* can degrade patulin directly [21], but the degradation mechanisms are unknown. In this paper, we investigated the possible mechanisms involved in degradation of patulin by antagonistic yeast using *P. caribbica* as the model organism.

The cell-free filtrate of *P. caribbica* decreased the concentrations of the patulin at all tested times indicating that *P. caribbica* might have produced certain intracellular enzymes which degraded the patulin. These results are similar to the degradation of OTA by the cell-free supernatant of *Bacillus subtilis CW 14* [33]. The functions of these enzymes were, however, short-lived as the degradation declined after 12 h. Moreover, the patulin may have reacted with sulfhydryl containing amino acids or proteins, thereby decreasing its concentration [17].

Co-incubation of heat-killed and live cells of *P. caribbica* with patulin respectively showed that the inactive yeasts could not reduce the concentration of the patulin, while the live cells significantly reduced the concentration of the patulin (Figure 2). This finding is in line with Dong et al. [34] who reported that the live yeast *Kodameae ohmeri* could degrade patulin. In addition, significant differences in the reduction of patulin content was observed between viable and heat-killed cells. The results showed that the degradation of patulin requires the presence of live yeast cells.

Fifty-one percent of patulin was absorbed by dead yeast cells of *R. paludigenum* [28]. However, the test on the role of *P. caribbica* in patulin absorption indicated that patulin was not detected in the broken yeast cells and the cell wall. These results showed that there are other mechanisms of patulin reduction other than absorption by the yeast cells.

The test on the effects of cycloheximide on *P. caribbica* degradation of patulin, demonstrated that the *P. caribbica* without the cycloheximide decreased the patulin concentration throughout the entire duration of the experiment and after 36 h the patulin could not be detected. On the contrary, *P. caribbica* containing the cycloheximide decreased the patulin concentration sharply during the first 12 h. These results are in agreement with the findings of Zhang et al. [35] who used cycloheximide combined with *S. cerevisiae* to degrade ZEN at time 0 h, while addition of cycloheximide at 12 h significantly slowed down degradation. Moreover, cycloheximide inhibits protein (enzymes) synthesis in eukaryotes. These results suggest that enzymes produced by antagonistic yeasts play an important role in degradation of patulin.

As reported, the process of patulin degradation is an enzymatic reaction [22,24,34]. This confirmed our findings in which the supernatant from the *P. caribbica* degraded the patulin regardless of whether the yeast cells were filtered out or not. This is an indication that the yeast may have synthesized some extracellular enzymes in the supernatant which was involved in the degradation.

Additionally, Folger [36] reported that patulin can be degraded by yeast protein extract. *R. paludigenum* was observed to have degraded patulin through the activities of its intracellular enzymes, and the enzymes were induced by the patulin [28]. In this way, the intracellular enzymes of *P. caribbica* alone and the *P. caribbica* induced with patulin were extracted and their ability to degrade patulin was confirmed. Our results showed that the intracellular enzymes of the *P. caribbica* induced with patulin contained the enzymes which were responsible for the degradation of patulin.

Ianiri, Idnurm, Wright, Durán-Patrón, Mannina, Ferracane, Ritieni, and Castoria [30] in their quest to determine the relevant genes responsible for patulin degradation used *A. tumefaciens* T-DNA delivery system. They isolated 13 mutants which were affected in patulin degradation, some exhibited hypersensitivity to patulin. However, the slow patulin degradation observed for the mutants was proven not to be the inactivation of genes encoding enzymes directly involved in the patulin degradation pathway, but to the loss of functions of genes involved in resistance to patulin-induced stresses. No degradation pathway was found. In this study, the differentially expressed proteins of *P. caribbica* induced by the patulin showed that the basic metabolic proteins of *P. caribbica* were affected. Response patterns of *P. caribbica* to patulin are complex, as the differentially abundant proteins were involved in multiple metabolic pathways. The cytoplasm of eukaryotic cells, whether chemical, thermal, or in the form of misfolded or aggregated protein, triggers a complex biological response referred to as the heat shock response [37,38,39,40]. Hsp70 is a molecular chaperone belonging to heat-shock protein family. It is a stress-inducible member, in contrast to Hsc70 expressed constitutively. Hsp70 plays an important role in the folding and assembling of newly synthesized proteins, refolding of misfolded proteins, aggregation of proteins, and membrane translocation of organelles. It also secretes proteins and controls the activities of regulatory proteins [41,42,43,44]. The more concentration indicated that Hsp 70 was produced during the degradation of patulin by *P. caribbica*. Phosphomannomutase (PMM, EC 5.4.2.8), catalyzing the interconversion between mannose-6-phosphate and mannose-1-phosphate, is an essential and conserved enzyme in eukaryotic organisms [45,46]. Mannose-1-phosphate is necessary for synthesizing the vital cellular metabolite GDP-mannose, which plays a crucial role in the formation of polysaccharide chains required for the glycosylation of protein and lipid molecules [47,48]. The mannan oligosaccharide is composed of mannose and glucose oligosaccharides, one of the main active ingredients of the cell wall of yeasts. It can absorb mycotoxins. So PMM may be an important enzyme in the degradation of patulin by *P. caribbica*. Most of the differentially expressed proteins are related to basic metabolism, such as acetyl-CoA hydrolase, isocitrate lyase, citrate synthase, and enolase 1.

## 4. Conclusions

In conclusion, the degradation of patulin by *P. caribbica* is an enzyme catalytic process, and these enzymes were induced by patulin. In the future, we will focus on the identification of these enzymes which are involved in patulin degradation and the degradation products of the patulin.

## 5. Materials and Methods

### 5.1. Antagonist and Growth Conditions

*P. caribbica* was isolated from soils of an unsprayed orchard (the central shoal of Yangtze River, Zhenjiang, Jiangsu Province, China) by our research team. Sequence analysis of the 5.8 S internal transcribed spacer (ITS) ribosomal DNA (rDNA) region of the yeast found that it was *P. caribbica* [49,50]. *P. caribbica* has been shown to be safe in animal testing, such as physiological, acute toxicity and Ames test [51]. *P. caribbica* isolates were maintained at 4 °C on nutrient yeast dextrose agar medium (NYDA-0.8% nutrient broth, 0.5% yeast extract, 1% glucose and 2% agar) (Sangon Biotech, Shanghai, China). Liquid cultures of the yeast were grown in 250-mL Erlenmeyer flasks containing 50 mL of nutrient yeast dextrose broth (NYDB) which had been inoculated with a loop of the culture. Flasks were incubated on a rotary shaker at 190 rpm for 48 h at 28 °C. Following incubation, cells were centrifuged at 6000× *g* for 10 min and washed twice with sterile distilled water. Cell pellets were re-suspended in sterile distilled water and adjusted to an initial concentration before being adjusted to the concentration required for different experiments.

### 5.2. Preparation of Stock Standard Solutions of Patulin

The patulin (Sigma-Aldrich, St. Louis, MO, USA) was prepared in accordance with the method described by MacDonald et al. [52], with some modifications. The patulin standard working solution (20 μg/mL) was diluted 10-fold with acetate buffer (pH 4.0). The samples were filtered through a 0.22 μm Wondadisc NY organic filter (SHIMADZU, Kyoto, Japan), and subjected to HPLC analysis. A standard curve of the patulin (μg/mL) was generated.

### 5.3. HPLC-UV Analysis of Patulin

Agilent 1100 series system (Agilent, Santa Clara, CA, USA) was used to analysis the patulin. The analytical column used was Zorbax, SB-C18 250 × 4.6 mm 5 μm (Agilent, Santa Clara, CA, USA). The mobile phase composed of water and CAN (9:1, *v*/*v*) that was set at 1 mL/min. The UV detection was performed at 276 nm. Data collection and subsequent processing were performed using Gilson Unipoint software 5.0 (Gilson, Inc, Middleton, WI, USA).

### 5.4. Efficacy of Cell-Free Culture Filtrate of P. caribbica on the Detoxification of Patulin

One milliliter culture medium of *P. caribbica* (1 × 10^8^ cells/mL) was added to 50 mL NYDB in 250-mL Erlenmeyer flasks. Flasks were incubated on a rotary shaker at 180 rpm at 28 °C for 20 h. Following incubation, cells were centrifuged at 6000× *g* for 10 min. Then cell suspensions were filtered through micro pore (0.22 μm) to obtain the cell-free filtrate. Twenty-five mL cell-free filtrate of *P. caribbica* and NYDB medium (control) were each added into 150-mL Erlenmeyer flasks containing patulin at the concentration of 20 μg/mL, respectively. Samples were taken every 6 h and centrifuged at 7000× *g* for 5 min. The supernatants were filtered through a 0.22 μm filter and HPLC analysis was performed to determine the patulin contents. Every group had three replicates and the experiment was replicated twice.

### 5.5. Efficacy of Viable and Heat-Killed P. caribbica Cells on the Reduction of Patulin

The yeasts cells were inactivated in water at 100 °C for 15 min. Two mL of the live *P. caribbica* (1 × 10^8^ cells/mL), the heat-killed cells, and sterile distilled water (control) were added to 25 mL NYDB containing 20 μg/mL of patulin in 150 mL Erlenmeyer flasks, respectively. Samples were taken at every 6 h, centrifuged at 7000× *g* for 5 min. The supernatants were filtered through a 0.22 μm filter, and subjected to HPLC analysis. Every group had three replicates and the experiment was replicated twice.

### 5.6. Absorption of Patulin by P. caribbica Cells

One milliliter suspension of *P. caribbica* (1 × 10^8^ cells/mL) was added to 25 mL NYDB and 20 μg/mL of patulin in 150 mL Erlenmeyer flasks. The samples were incubated in a rotary shaker at 180 rpm for 20 h at 28 °C. Following incubation, cells were centrifuged at 6000× *g* for 10 min and washed twice with sterile distilled water to remove the patulin in the supernatant. The yeasts were sonicated at 1000 hz for 20 min. Fifteen mL of ethyl acetate was added to broken yeast cells, vortexed for 60 s and the upper layer was transferred into a separate funnel (125 mL). This step was repeated thrice and then 5 mL of 1.4% (*w*/*v*) Na_2_CO_3_ was added to the ethyl acetate layer and vigorously mixed for 2 min. The upper ethyl acetate layer was dried in a vacuum at 40 °C in a rotary evaporator. Soon after, 1 mL acetate buffer (0.2 mol/L, pH 4) was added and vortexed until it dissolved completely. The sample was filtered through a 0.22 μm Wondadisc NY organic filter (SHIMADZU, Kyoto, Japan), and analyzed in HPLC machine. Every group had three replicates and the experiment was replicated twice.

### 5.7. Effects of Cycloheximide on Degradation of Patulin by P. caribbica

One milliliter suspension of *P. caribbica* (1 × 10^8^ cells/mL) was added to 25 mL NYDB and 20 μg/mL of patulin in 150 mL Erlenmeyer flasks. Treatments were as follows: (1) 5 μg/mL cycloheximide with patulin; (2) Addition of patulin into cycloheximide after 6 h; (3) only patulin (Control). Samples were taken at every 6 h, and centrifuged at 7000× *g* for 5 min. The supernatants were filtered through a 0.22 μm filter, and subjected to HPLC analysis to determine the patulin content. Every group had three replicates and the experiment was replicated twice.

### 5.8. Effects of P. caribbica Supernatant on Patulin Degradation

Two milliliter suspension of *P. caribbica* (1 × 10^8^ cells/mL) was added to 25 mL NYDB and 20 μg/mL of patulin. After incubating for 6 h, one group was filtered twice through 0.22 μm filter to remove all the cells and the group was not filtered. Samples were taken every 6 h and centrifuged at 7000× *g* for 5 min. The supernatants were filtered through a 0.22 μm filter and analyzed using HPLC. Every group had three replicates and the experiment was replicated twice.

### 5.9. Degradation of Patulin by Intracellular Enzymes

*P. caribbica* (10^8^ cells/mL) was incubated in NYDB and NYDB containing 20 μg/mL patulin in a rotary shaker at 180 rpm at 28 °C. After 20 h of incubation, the supernatants and the cells were centrifuged. The cells were washed with phosphate buffer (50 mM, pH 7.0) thrice. The wet cells were quickly ground in mortar using pestle with liquid nitrogen added and then suspended in 10 mL phosphate buffer. After 30 min in ice, the samples were centrifuged at 13,000× *g* for 10 min at 4 °C and the supernatant was collected. Afterwards, 100 µg of patulin was added to 5 mL of the yeast cells was amended with the patulin and those that were not amended. Every group had three replicates and the experiment was replicated twice.

### 5.10. Protein Sample Preparation

Liquid cultures of the yeasts were grown in NYDB and NYDB + 20 μg/mL patulin as described above under the section Antagonist and Growth Conditions’. The protein samples were prepared as described by Li et al. [53] with some modifications. After 24 h, the yeast cells were harvested from the NYDB and the NYDB + patulin, and centrifuged at 10,000× *g* for 10 min (4 °C). The cells were washed with cold distilled water each time after centrifugation to remove residual medium. Subsequently, the samples (yeast cells) were ground into fine powder with a mortar and pestle. The powder was transferred into a 50-mL tube. Thereafter, 10 mL TE buffer (10 mM Tris-HCL, pH 8.0, 1 mM EDTA, and 1 mM PMSF), 50 μg of RNase A and 200 μg of DNase I was added and incubated at 4 °C for 30 min. The samples were centrifuged at 11,000× *g* for 20 min (4 °C), then the supernatant was added to two volumes of 20% TCA/acetone (−20 °C pre-cooled at least 30 min), vortexed, and incubated at −20 °C for 12–16 h. Following incubation, the sample was centrifuged at 11,000× *g* for 20 min (4 °C) and the supernatant was discarded. The pellets were centrifuged at 11,000× *g* for 5 min (4 °C), washed with acetone (−20 °C pre-cooled). The pellets were air-dried at room temperature to remove residual acetone. Then solubilized in lysis buffer containing 2 M thiourea, 7 M urea, 4% (*w*/*v*) CHAPS, 65 mM DTT, 0.2% (*w*/*v*) Bio-Lyte (Bio-Rad, Hercules, CA, USA). Protein samples were kept at −80 °C until use. The protein concentration was determined according to Bradford’s method using bovine serum albumin as standard [54].

### 5.11. 2-DE and Image Analysis

Two-dimensional electrophoresis (2-DE) and image analysis were performed as described by Wang et al. [55]. The first dimension electrophoresis was carried out on a 17-cm IPG strip (pH 3–10, Bio-Rad, Hercules, CA, USA). The strip was rehydrated for 1 h in the rehydration solution containing 2 M thiourea, 7 M urea, 4% (*w*/*v*) CHAPS, 65 mM DTT, 0. 2% (*w*/*v*) Bio-Lyte, and about 400 μg sample protein in a re-swelling trough. The rehydrated strip was then subjected to electrophoresis in the first dimension. After isoelectric focusing, the strip was equilibrated in two steps in the SDS equilibration stock solution consisting 50 mM Tris-HCl buffer, 6 M urea, 20% (*v*/*v*) glycerol and 2% (*w*/*v*) SDS supplemented with 2% (*w*/*v*) DTT, and 2.5% (*w*/*v*) iodoacetamide, respectively. The second dimension was run on a 12.5% polyacrylamide gel using the Multiphor system (Amersham Biosciences, Amersham, UK). The conditions were 1 W per strip for 1 h followed by 15 W per strip until bromophenol blue reached 0.5 cm above the bottom. To estimate the molecular weights of the protein spots, marker proteins were also separated together with the *P. caribbica* proteins. After electrophoresis, gels were visualized by Coomassie Blue stain. The stained gels were scanned and analyzed using PDQuest software (version 7.4, Bio-Rad, Hercules, CA, USA). Proteins that increased two-fold at one point after treatment, as well as exhibited the same expression pattern among the replicates, were considered as significant and reproducible change proteins. The proteins were subsequently identified. At least three biological replicates were performed for each treatment.

### 5.12. Protein In-Gel Digestion and Identification

Differentially expressed protein spots were excised from the gel and were washed twice by distilled H_2_O, then destained with 50 mM NH_4_CO_3_/CAN solution. Afterwards, they were washed with 25 mM NH_4_CO_3_, 50% CAN until the gels became white, then vacuum drained for 5 min. Two μL trypsin (10 μg/μL) (Sigma–Aldrich, St. Louis, MO, USA) was incubated at 4 °C for 30 min, then 10 μL 25 mM NH_4_CO_3_ was added. The gels were incubated overnight at 37 °C. The supernatant was collected for MS analysis [56]. Proteins were identified by MALDI-TOF/TOF and database query. The peptide solution was analyzed using MALDI TOF/TOF mass spectrometer (Ultraflex III, Bruker-Daltonics, Bremen, Germany). MS/MS spectra were analyzed using the FlexAnalysis 3.0 (Bruker Daltonics GmbH, Bremen, Germany). The resulting monoisotopic peptide masses were queried against the protein database in NCBInr using MASCOT version 2.3 software (Matrix Science, Franklin, UK) with the following search parameters: all entries, trypsin, up to one missed cleavage, carbamidomethyl (C), oxidation (M), and Gln-Pyro-glu, peptide tolerance 0.3 Dal, mass value MH+, and monoisotopic [37].

### 5.13. Statistical Analysis

The data were analyzed by the analysis of variance (ANOVA) in the statistical program SPSS/PC version 17.0, (SPSS Inc. Chicago, IL, USA) and the Duncan’s multiple range test was used for means separation. In addition, when the group of the data was two, the independent samples *t* test was applied for means separation. The statistical significance was assessed at the level *p* < 0.05.

## Figures and Tables

**Table 1:** Proteins identified with PMF.

Molecular Function	Spot	Protein Name	NCBI Accession	Mass	PI	Species	Score	Sequence Coverage (%)	Number of Mass Values Matched
Serine-type endopeptidase activity	2	Chymotrypsinogen A	gi|117615	26,220	8.52	Bos taurus	206	16	4
Serine-type endopeptidase activity	21	Chymotrypsinogen	gi|117616	26,309	4.99	Bos taurus	110	6	1
Isocitrate lyase activity	4	Isocitrate lyase	gi|146413757	61,937	6.31	Meyerozyma guilliermondii ATCC 6260	124	5	2
Isocitrate lyase activity	8	Isocitrate lyase	gi|146413757	61,937	6.31	Meyerozyma guilliermondii ATCC 6260	429	12	5
Isocitrate lyase activity	11	Isocitrate lyase and phosphorylmutase	gi|344232420	62,282	6.78	Candida tenuis ATCC 10573	67	5	2
Citratesynthase activity	6	Citrate synthase	gi|146421975	43,995	6.25	Meyerozyma guilliermondii ATCC 6260	119	5	2
Acetyl-CoA hydrolase activity	10	Acetyl-CoA hydrolase	gi|146417797	58,385	5.96	Meyerozyma guilliermondii ATCC 6260	222	6	2
Phosphopyruvate hydratase activity	15	Enolase 1	gi|146415384	46,951	5.42	Meyerozyma guilliermondii ATCC 6260	117	3	1
Phosphomannomutase activity	17	Phosphomannomutase	gi|146423739	28,678	5.26	Meyerozyma guilliermondii ATCC 6260	361	19	5
Stress response	20	Heat shock protein SSB1	gi|146420661	66,421	5.29	Meyerozyma guilliermondii ATCC 6260	260	10	5
Stress response	22	Heat shock protein 70 2	gi|146413777	70,177	5.04	Meyerozyma guilliermondii ATCC 6260	951	17	12
Unclassified	12	DEHA2F04796p	gi|50423973	35,926	6.24	Debaryomyces hansenii CBS767	61	7	2
Unclassified	13	Conserved hypothetical protein	gi|146417765	34,916	7.17	Meyerozyma guilliermondii ATCC 6260	219	13	3
Unclassified	14	Hypothetical protein PGUG_05640	gi|146413298	65,256	5.85	Meyerozyma guilliermondii ATCC 6260	170	5	2
Unclassified	16	DEHA2G14058p	gi|50427089	47,210	5.28	Debaryomyces hansenii CBS767	197	7	2
Unclassified	5	Hypothetical protein PGUG_05024	gi|146414197	32,118	7.77	Meyerozyma guilliermondii ATCC 6260	122	10	2
Unclassified	9	DEHA2D06160p	gi|50420381	54,282	5.68	Debaryomyces hansenii CBS767	133	8	2
Unclassified	23	Conserved hypothetical protein	gi|146416825	44,146	5.33	Meyerozyma guilliermondii ATCC 6260	280	13	4
Unclassified	24	Hypothetical protein PGUG_00755	gi|146422888	20,317	5.45	Meyerozyma guilliermondii ATCC 6260	84	6	1
Unclassified	25	Hypothetical protein PGUG_04067	gi|146414736	34,234	6.64	Meyerozyma guilliermondii ATCC 6260	104	11	3
Unclassified	24	Hypothetical protein PGUG_03175	gi|146418962	62,015	5.21	Meyerozyma guilliermondii ATCC 6260	272	12	4
Unclassified	29	Hypothetical protein PGUG_00646	gi|190344794	78,744	8.19	Meyerozyma guilliermondii ATCC 6260	296	11	6
Unclassified	30	Hypothetical protein LOC100448380	gi|297701166	105,979	5.19	Pongo abelii	85	3	3
Unclassified	31	Hypothetical protein PGUG_03973	gi|146414548	38,139	5.53	Meyerozyma guilliermondii ATCC 6260	127	5	1
Unclassified	32	Hypothetical protein PGUG_01788	gi|146420321	37,067	5.56	Meyerozyma guilliermondii ATCC 6260	109	9	2
Unclassified	33	Hypothetical protein PGUG_00294	gi|146421948	35,991	5.22	Meyerozyma guilliermondii ATCC 6260	88	8	2
Unclassified	1	Chain Z	gi|230350	24,662	8.23	Bos taurus	316	29	6
